# Flexion Relaxation and Its Relation to Pain and Function over the Duration of a Back Pain Episode

**DOI:** 10.1371/journal.pone.0039207

**Published:** 2012-06-15

**Authors:** Raymond W. McGorry, Jia-Hua Lin

**Affiliations:** Liberty Mutual Research Institute for Safety, Hopkinton, Massachusetts, United States of America; University Medical Center Groningen UMCG, Netherlands

## Abstract

**Background:**

Relaxation of the erector spinae often occurs in healthy individuals as full trunk flexion is achieved when bending forward from standing. This phenomenon, referred to as flexion relaxation is often absent or disrupted (EMG activity persists) in individuals reporting low back pain (LBP).

**Methods and Results:**

Self-reported pain and disability scores were compared to EMG measures related to the flexion relaxation (FR) phenomenon by 33 participants with LBP at up to eight sessions over a study period of up to eight weeks. Fourteen participants served as a control group. In the protocol, starting from standing participants bent forward to a fully flexed posture, and then extended the trunk to return to standing position. A thoracic inclinometer was used to measure trunk posture. Surface electrodes located at the L2 and L5 levels recorded EMG amplitudes of the erector spinae. Ratios of EMG amplitudes recorded during forward bending to amplitudes at full flexion, and ratios of extension to full flexion were calculated. EMG amplitudes and their ratios were compared between control and LBP groups at the initial visit. No significant differences between groups were found except at the L5 location at full flexion. Correlations of the ratios to pain and function scores recorded in repeated sessions over the LBP episode also were compared between LBP group participants classified as having transient, recurrent or chronic symptoms. In another analysis participants were grouped by whether their symptoms resolved over the study period.

**Conclusions:**

The transient LBP group had significantly stronger correlations between pain and function to both ratios, than did those with more chronic LBP symptoms. Participants who experienced symptom resolution generally had stronger correlations of ratios to both pain and function than those with partial or no resolution. Improved understanding of these relationships may provide insight in clinical management of LBP.

## Introduction

The observation of electrical silence of the erector spinae (ES) at full trunk flexion was first referred to as flexion relaxation (FR) by Floyd and Silver [1]. Though often studied since, the exact mechanism for FR is not definitively known. One proposed mechanism may be stretch reflex inhibition, a reflexive contraction orchestrated by the muscle spindle in response to passive longitudinal stretching [1], [2]. The lumbodorsal fascia and other ligaments might provide the necessary supporting moment for the trunk, reducing the necessity of active muscular contraction to maintain the fully flexed posture [3]. Adams et al. suggested that when the ES is electrically silent at full trunk flexion, passive tension of the muscle tissue could provide some resistance to trunk moment [4].

The literature suggests that FR of the lumbar ES at full trunk flexion is observed in the majority of healthy individuals without back pain, though substantial variability in the behavior exists, likely due to individual differences such as anthropometric variation as well as differences in protocols (e.g. posture, electrode placement) [1], [5]–[10]. It has also been observed that FR may vary with change in the speed of the flexion/extension motion, prolonged static flexion, muscle fatigue, external load application, and with compound motions [6], [9]–[14]. The sEMG amplitude of the ES during trunk extension against gravity (concentric) is typically greater than during the eccentric trunk flexion phase [8], [15].

One method reported for quantifying FR, to best allow comparison of measures repeated over time or between individuals, is to calculate the ratio of the sEMG amplitude of the ES during the trunk flexion phase to that recorded at full static flexion. This technique, commonly referred to the “flexion relaxation ratio,” or similar terminology, was first reported by Sihvonen et al. [7], and has been widely adopted as a method for quantifying FR [8], [16]–[22].

In many studies, FR was absent or significantly impaired (sEMG activity persists at full trunk flexion) in those with low back pain (LBP). Absence or impairment of FR has been reported to vary from 41% of cases (in a population of subjects with a history of LBP, but pain free at the time of testing) to as many as 100% of subjects with active LBP [5], [7]. Geisser et al. in their meta-analysis reported that FR could discriminate between individuals with and without LBP [18]. Using various FR-related measures of trunk flexion/extension in standing, several reports show differences between normal (pain-free) and LBP groups [7], [8], [11], [16], [23].

FR may vary with severity and duration of LBP symptoms, and a re-establishment of FR may reflect clinical improvement. The literature shows that, in some but not all individuals for whom FR was absent (electrical silence did not occur at full flexion) while experiencing an episode of LBP, FR was reestablished when their symptoms resolved [1], [24]–[26]. Paquet et al. in their report on the relationship of muscle activity and lumbar/pelvic coordination concluded “…the lack of relaxation of the ES muscle may be associated with perturbation of movement patterns and the duration of symptoms” [11]. More recently, several studies reported results of pre-treatment – post-treatment evaluations of exercise or functional restoration programs with patients with chronic LBP in a tertiary care setting. These results suggest that some measures related to the FR phenomenon had some ability to discriminate functional improvement in patients with chronic LBP following back rehabilitation programs [21], [27]–[30]. While there is preliminary evidence that FR can be restored, whether partially or fully, more research is needed to evaluate longitudinal changes in FR over time in relation to symptoms.

While the studies cited above have focused on FR-related measures as a method to distinguish individuals with and without LBP, or as a method for documenting or guiding rehabilitation of those with chronic LBP, few studies have attempted to correlate FR with measures of self-reported pain and/or disability [22], [24], [31]. Further, there are no reports in the literature of the nature of, or changes to FR-related measures using repeated measures over the course of an LBP episode. Toward the goal of improved understanding of how FR changes with changes in pain and function over time, the present study will investigate how these factors relate among a community sample of individuals during a prospective study conducted over the natural course of an episode of nonspecific LBP. These measures and their relationships will also be compared to those obtained in a symptom-free control group. The correlation of FR related measures to self-reported function and the intensity of back pain were evaluated over the a period of up to eight weeks, and the results are compared for participant grouping based on symptom history, and also based on groupings based on resolution of pain symptoms. The results should provide some clinical insight for the practitioner in treatment of the individual experiencing an episode of non-specific LBP.

## Methods

### Ethics Statement

All research involving human participants was approved by the institutional review board of the Liberty Mutual Research Institute for Safety. Written informed consent was obtained, and all data was de-identified, kept confidential and analyzed anonymously. The clinical investigation was conducted according to the principles expressed in the Declaration of Helsinki.

### Participants

Men and women experiencing a LBP episode were recruited by means of advertisements posted at local clinicians' offices (physical therapists, chiropractors, and physicians) and by newspaper advertisement to participate in a maximum eight-week clinical study. The inclusion criteria for the study were that potential participants be 18 to 65 years of age and presently experiencing LBP. Participants were included whether it was their first experience with LBP, or if they were experiencing a recurrence. The purpose and protocol for the study was explained to all respondents, and those that expressed interest completed a medical history form and were interviewed and examined by a health care provider. Medical exclusion criteria were: major structural abnormalities, significant neurological deficits or evidence of severe nerve root compression, active systemic, inflammatory, musculoskeletal or neoplastic disease or history of previous back surgery. An additional exclusion criterion was having an active worker's compensation claim or related litigation pending, to avoid any potential confounding due to medico-legal concerns. Thirty-four individuals meeting the study criteria for nonspecific back pain were enrolled and assigned to the LBP group. One participant withdrew after the initial visit. Thirty-three participants belonging to the LBP group completed the multi-session protocol.

Eighteen participants were recruited for the Control group. The selection criteria were that participants be 18 to 65 years of age, in good health and had no significant history of back pain. Fourteen from the Control group performed a single session of the experimental protocol. The remaining four participants completed four additional sessions (five sessions in total) performing the experimental protocol at two-week intervals over an eight-week period. This multi-session subset served to provide some indication of the inherent variability in the dependent measures. Participant demographic information for the two groups was collected and is presented in Table 1.

**Table 1:** Participant demographics.

	n	Gender	Age	Height	Weight
			(yr)	(cm)	(kg)
**Control**	**18**	10 M, 8 F	35.2 (9.4)	168.4 (10.5)	69.7 (11.7)
**LBP**	**33**	17 M, 16 F	40.5 (12.8)	168.9 (10.4)	75.0 (15.3)

### Experimental design

A within-subject repeated measures design was used. The two levels of pain status were LBP and Control (no-LBP). The dependent variables were trunk inclination and sEMG amplitude at four locations on the erector spinae.

### Equipment

#### Trunk inclination

Trunk kinematics were evaluated using an electronic inclinometer (Model #N4, Seika Corp., Tokyo, Japan). The inclinometer was attached to an appropriately sized adjustable harness/vest (small, medium, large). The inclinometer was located dorsally at the mid-thoracic region overlying the sixth thoracic spinous process, and this orientation allowed measurement of gross trunk flexion/extension.

*Surface EMG –* Four differential surface electrodes and an amplification and conditioning system (Bagnoli-8 EMG System, Delsis Corp., Boston, MA) were used in this study. The electrodes, with an inter-electrode distance of 1 cm, had onboard amplification with a frequency response of 20 to 450 Hz, and a common mode rejection ratio of 92 dB. A gain of 1000 was used. Four sEMG signals and the inclinometer output were sampled at 1000 Hz and stored in computer memory.

### Subjective measures (LBP group only)

#### Daily Pain Score

A numerical pain rating scale (NPRS) was used to quantify the intensity of back pain. Participants rated their pain a 0 to 10 scale, where the anchors at 0 and 10 were “no pain” and “the worst pain imaginable”, respectively [32]. At the beginning of each experimental session participants rated the pain intensity they were feeling “right now.”

#### Functional Level

At the start of each experimental session participants also reported on their function in daily activities today using the clinically validated Back Pain Functional Scale (BPFS) [33]. This questionnaire rates impairment on a 0 to 5 scale, with each point anchored by a functional rating ranging from “unable to perform activity” to “no difficulty” for 12 activities, providing a functional continuum from 0 representing total dysfunction to 60, normal function.

### Experimental protocol

At the initial experimental session with the LBP participants, the experimental nature of the measurements was discussed and that the protocol was not related to treatment was reinforced. Participants were then educated in pain scoring, and rating function using the BPFS. Participants from both groups were treated as follows. While standing, the sEMG sites were located and marked at the level of L2 and L5 spinous processes, 2.5 cm to the left and right of midline. The electrode placement was selected for consistency with previous reports in the FR literature [e.g. 16, 19, 22, 27]. For the LBP group and the four members of the Control group that participated in the eight-week protocol, the electrode locations and skin landmarks were transferred to a transparent plastic film to permit consistent repositioning during subsequent sessions. The four electrode sites were then prepared with an alcohol scrub, and shaved when necessary. Electrodes were oriented along the long axis of the muscle and attached using skin tape. The reference electrode was placed over the right clavicle. The harness with the inclinometer was donned so that the inclinometer was maintained firmly over the posterior midline at the mid-thoracic level.

**Figure 1:**
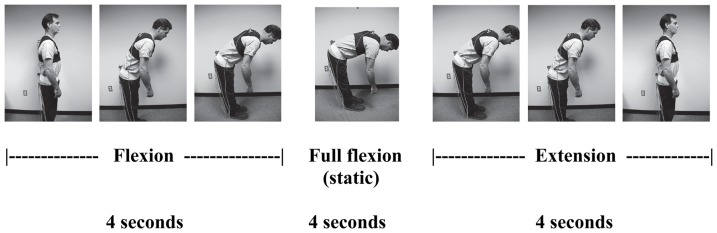
The experimental motion in standing illustrating the trunk flexion, static flexion, and extension phases, four seconds each.

The experimental task, a flexion/extension motion starting in standing, was paced by a computer running a custom data acquisition program that produced a series of audible beeps. With feet shoulder-width apart and looking forward the participant was instructed to move in response to the audible cues, keeping the knees straight but not locked and the arms hanging freely, while slowly flexing forward to full flexion over a four-second period, pausing for four seconds at full trunk flexion, and then returning to the upright starting position during a four-second trunk extension period. This protocol is typical of those used in studies of flexion relaxation [e.g. 1, 28, 30]. Figure 1 demonstrates the timing of the experimental task. Three replications of the motion were performed. The first trial was treated as practice and was omitted from analysis to minimize transient effects related to muscle warm-up, or stretching. Data from the last two replications were used in the subsequent analysis.

LBP group participants were scheduled for eight visits distributed over an eight week period scheduled twice a week for the first two weeks, once a week for the third and fourth weeks and once each in the sixth and eighth weeks. A stopping criterion was used for LBP participants whose pain resolved during the course of the experimental protocol. When participants reported a pain score of “0” at two consecutive sessions, participation was discontinued. When the experimental session was scheduled to coincide with a treatment session at a clinic, ratings and measurements were made prior to treatment, to minimize confounding by the effect of the treatment. The subset of four Control participants performed the protocol at five biweekly sessions.

### Data reduction

The inclinometer signal for each trial, filtered with a 4th order zero-lag low pass Butterworth filter with an 8 Hz cutoff, was displayed on the computer screen. Using a custom software program one research team member marked four inflection points of the inclinometer tracing dividing the experimental task into three phases: the flexion phase (FL), the static fully flexed or flexion relaxation (FR) phase and the extension phase (EX). The sEMG signals were RMS filtered with a 100 ms centered window. Within the FL and EX phases the peak amplitude of each of the four EMG sites was determined, and the mean for a one-second window about the peak was taken as the sEMG amplitude value. The value for the FR phase was calculated by taking the mean of a one-second window about the midpoint between the end of FL phase and the beginning of EX phase. The mean was taken for each phase for the two replications recorded at each session. The mean was then taken for the means for the left and right L2 sEMG, and the left and right L5 sEMG, yielding six measures used in the analysis: L2 and L5 amplitude during flexion (FL_L2_, FL_L5_), L2 and L5 amplitude during the flexion relaxation (FR_L2_, FR_L5_) and L2 and L5 amplitude during extension (EX_L2_ and EX_L5_).

#### Flexion relaxation ratios

**Figure 2:**
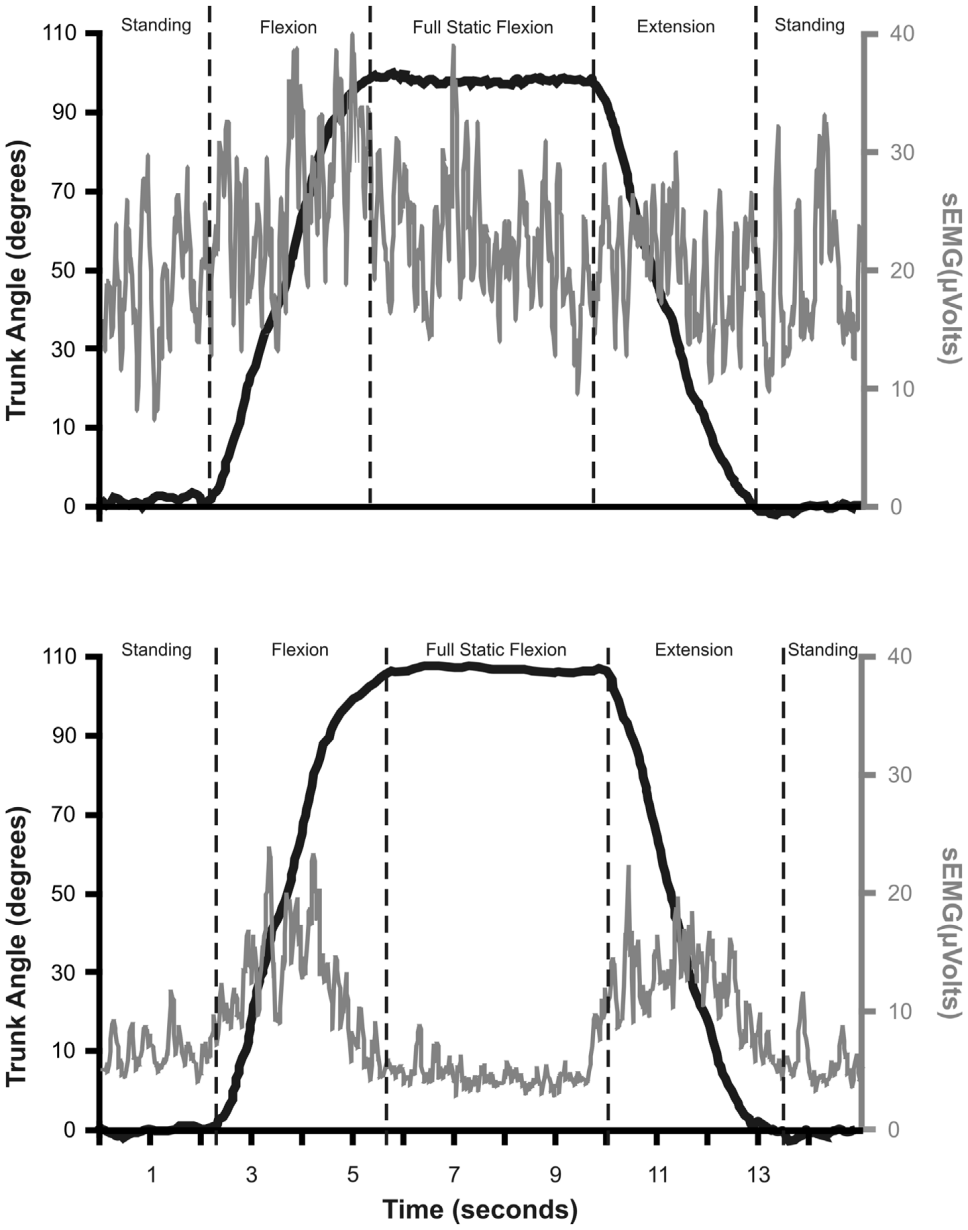
Trunk inclinometer and L5 sEMG tracings demonstrating different flexion relaxation states: 2a) absent flexion relaxation, 2b) normal flexion relaxation.

Calculating ratios of sEMG amplitudes between the phases of motion is a technique that allows normalization for repeated measures over time or for between-subject comparisons. The ratio of mean sEMG amplitude during the flexion movement to the mean amplitude during the FR phase for the L2 and L5 locations (FL-FR_L2_ and FL-FR_L5_, respectively) was calculated as previously described by Watson et al. and Ahern et al. [16], [23]. This methodology was also used with the mean amplitudes determined for the extension phase (EX-FR_L2_ and EX-FR_L5_). Higher ratios indicate relatively more flexion relaxation (less activation) of the erector spinae at full trunk flexion. As an example, Figure 2 provides graphs of L5 sEMG and trunk angular displacement recorded during the experimental task. When Figure 2a was recorded the participant reported pain and functional limitations. The FL-FR_L5_ ratio was 1.2, and the EX-FR_L5_ ratio was 0.9. Figure 2b was recorded at a subsequent session where the participant reported no LBP or functional limitation, and had FL-FR_L5_ and EX-FR_L5_ ratios of 2.7 and 2.3, respectively.

#### Group assignments

Group assignments were made for participants with LBP based on two factors. The first group assignments were based on their self-reported “pain history,” using a criteria proposed by Von Korff [34]. The three group assignments were Transient, Recurrent, and Chronic. Transient group assignment was for those experiencing first episode of LBP, and reporting having pain less than half the days of the past six months. The Recurrent group had a history of LBP greater than six months, but reporting pain on fewer than half the days during that period. The Chronic group reported LBP on greater than half the days of the past six months.

The second grouping was based on the degree of improvement in LBP symptoms, or “pain resolution”, reported over the period of study participation. Assignment to the Resolved group was for participants who reported a “0” pain score on the final day of their enrollment. Partial resolution designation was for participants who reported a pain score of “1” on the final visit indicating that the participant was not reporting full resolution, and at least some degree of pain is present. The unresolved group designation was applied those with a “2” or greater score at the final visit.

### Statistical analysis

One-way repeated measure ANOVAs were performed with the Control and LBP group initial visit data to test for differences in the four sEMG ratios. Spearman correlation coefficients were calculated for each participant between pairs of pain scores and the sEMG ratios, and between the BPFS function score and the sEMG ratios. Multivariate analysis of variance (MANOVA) was performed to test the effect of group divisions based on both pain history and pain resolution, on the sEMG measures. Tukey's post hoc analysis was employed if the effect was significant. The criterion selected for statistical significance was set at p = 0.05.

## Results

At the initial experimental session the 33 LBP group participants reported a mean (standard deviation) of 7.3 (7.5) year history of back pain. At the initial session the LBP group reported a pain score of 3.0 (1.6) and a function score of 43.9 (8.0) on the BPFS scale. LBP group participated in a total 248 study sessions. Twenty-seven participants completed the full eight-session protocol. Based on the stopping criteria (“0” pain scores for two consecutive visits), three participants completed seven sessions, one completed five sessions and two completed three sessions. These six participants remained pain-free for the remainder of the eight-week protocol. Six additional participants gave a “0” pain score on their final visit, for a total of 12 participants reporting resolution of their symptoms by the final visit. The gross trunk flexion range of motion (ROM) during the experimental task was 111.0° (17.0) and 112.8° (16.7) for the Control and LBP groups, respectively.

**Figure 3:**
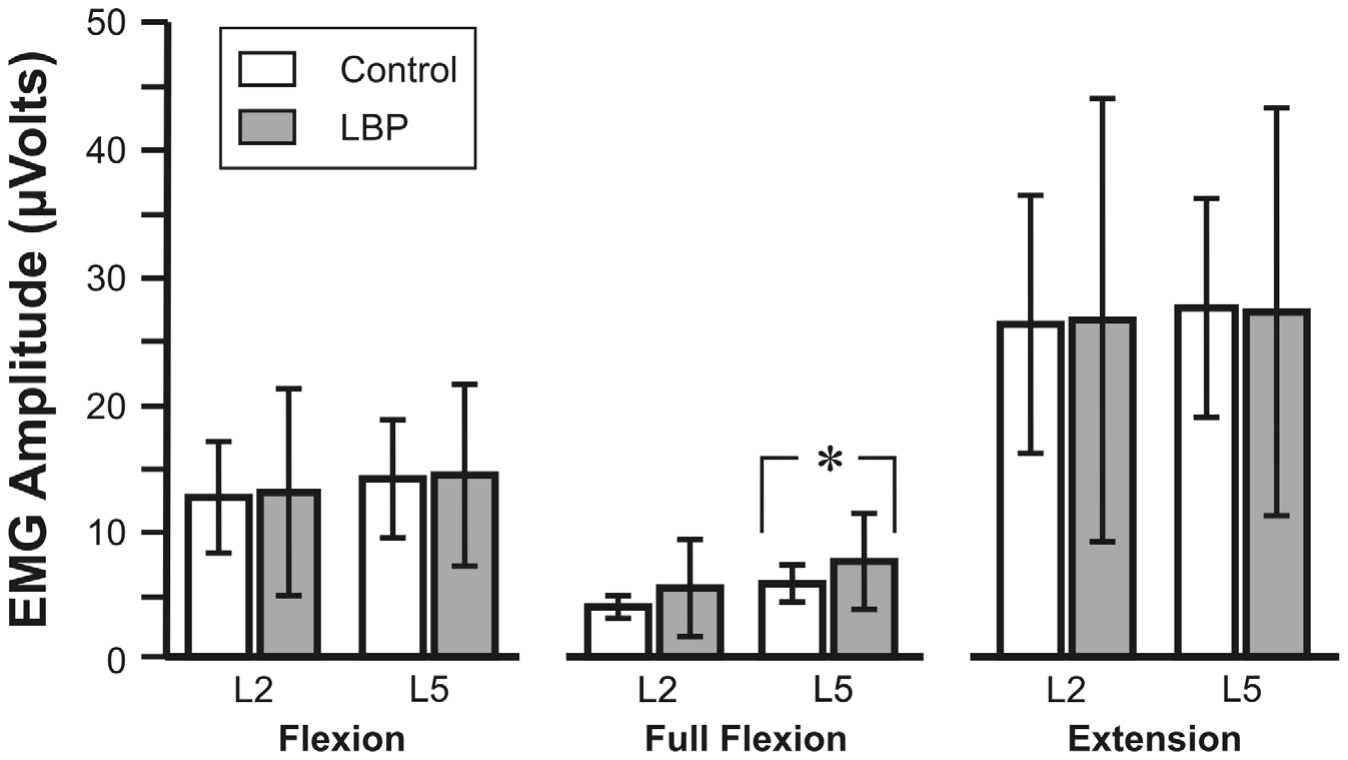
L2 and L5 sEMG amplitudes for the three phases at the initial visit. The asterisk indicates significant differences.

The sEMG amplitudes recorded at the two lumbar levels for the three phases of the experimental task are presented in Figure 3 for the Control and LBP groups at the initial visit. At the initial visit there was a significant difference between the LBP and Control groups in L5 sEMG amplitude during the FR phase, 7.7 (3.9) µV and 5.7 (1.5) µV, respectively. There were no other significant between-group differences in amplitudes at the initial visit. For both groups sEMG amplitudes were generally greater during the eccentric contraction of trunk flexion phase than the subsequent FR period. The mean amplitudes occurring during trunk extension when returning to the standing posture (concentric contraction) were generally greater than for the observed for the eccentric contraction of the initial flexion phase.

**Figure 4:**
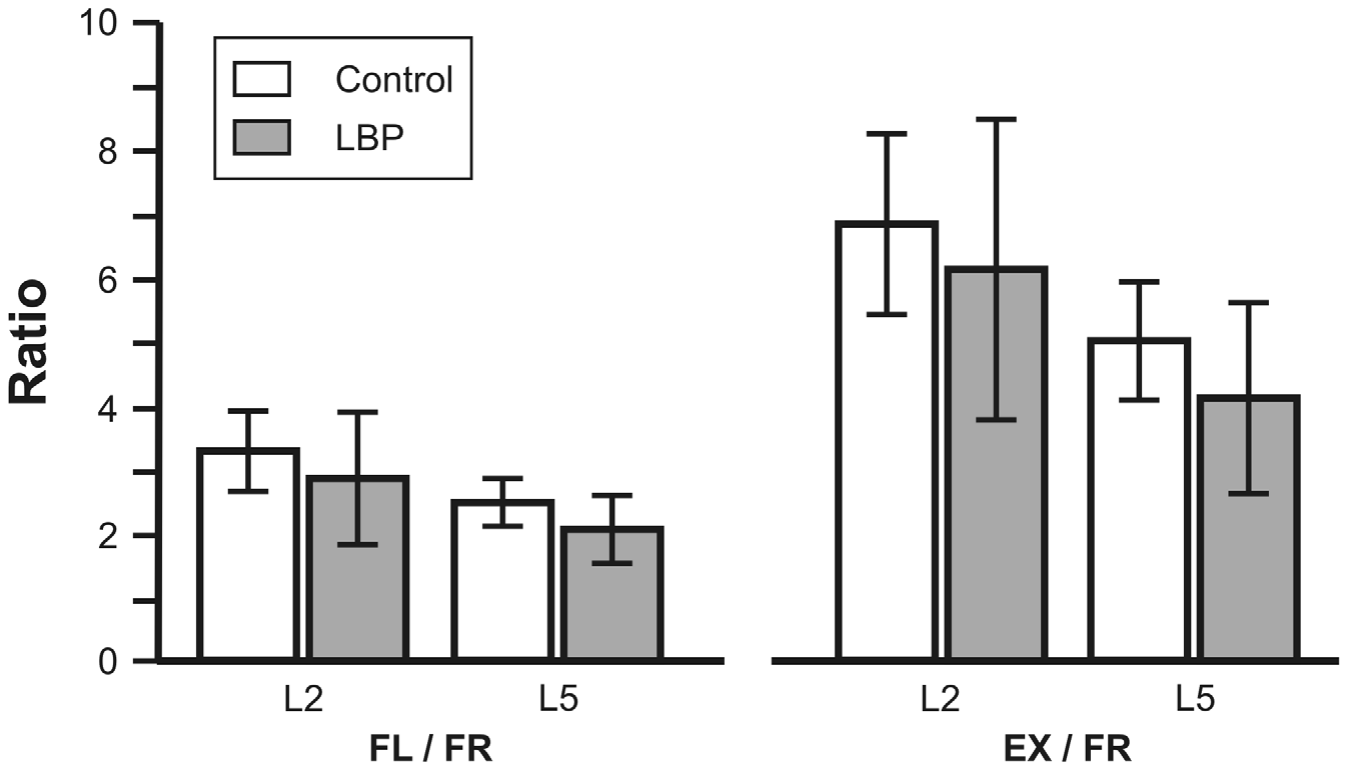
L2 and L5 FL-FR and EX-FR ratios for the three phases at the initial visit.

Figure 4 shows bar graphs of FL-FR_L2_ and FL-FR_L5_ and EX-FR_L2_ and EX-FR_L5_ ratios for the Control group and for LBP groups recorded at the initial visit. There were no significant differences in any of the sEMG ratios between LBP and Control groups at the initial visit. The data from the subgroup of Controls was evaluated to provide an indication of the variability of these ratios in repeated measures of pain-free individuals over time. Means of the coefficients of variation between the ratios calculated for each of the four participant's experimental sessions were FL-FR_L2_ = 0.48, FL-FR_L5_ = 0.33, EX-FR_L2_ = 0.20 and EX-FR_L5_ = 0.18.

ANOVAs were conducted to test for differences between sEMG ratios recorded at the initial visit and final visits for participant groupings based on characteristics of their pain experience. Grouping of participants based on pain history yielded assignment to the three groups in the following proportions: Transient (n = 8), Chronic (n = 7) and Recurrent (n = 18). ANOVA revealed no significant differences between any of the four sEMG ratios recorded on the first and last visits, for any of the three groups. The groupings based on pain resolution produced group assignment in the following proportions: Resolved (n = 12), Partial resolution (n = 8), Unresolved group (n = 13). For the Resolved group the difference in the FL-FR_L5_ ratio approached statistical significance (p = .06) between the first visit, 1.56 (0.68), and last visit, 2.04 (0.48), but no significant differences were found between the first and last visits for any of the four sEMG ratios recorded on, for any of the groups.

The Spearman correlation coefficients calculated between the sEMG ratios and the pain scores were generally negative, with increasing pain scores associated with decreasing ratios. Correlations between function ratings and ratios were generally positive, being indicative of more pronounced FR (and thus greater ratios) with improving function. Correlations between sEMG ratios and pain and function taken for the LBP population as a whole did not exceed 0.20, and no correlations were statistically significant. Correlations were generally stronger for some groups, in the analysis of groups based on history pain behavior and symptom resolution. Table 2 presents Spearman correlation coefficients calculated between the sEMG ratios and both pain scores and function ratings for the analysis of participants grouped based on pain history and on pain resolution.

**Table 2:** Mean (s.d.) Spearman correlation coefficients between pain scores and EMG ratings, and function ratings and EMG ratings, grouped by pain history and pain resolution criteria.

	FL-FR_L2_	FL-FR_L5_	EX-FR_L2_	EX-FR_L5_
**Correlation of Pain Score to sEMG ratio:**				
**Pain History**				
**Transient**	−0.35 (0.54)	−0.32 (0.50)^A^	−0.15( 0.67)	−0.41 (0.40)^A^
**Chronic**	0.03 (0.28)	0.26 (0.37)^B^	−0.04( 0.23)	0.09 (0.33)^B^
**Recurrent**	−0.09 (0.45)	−0.16 (0.34)	−0.16 (0.37)	−0.18 (0.34)
**Resolution of Pain**				
**Resolved**	−0.24 (0.54)	−0.36 (0.45)^A^	−0.10 (0.57)	−0.38 (0.37)
**Partial**	−0.16( 0.35)	0.06 (0.27)	−0.23 (0.24)	−0.08 (0.23)
**No Res**	−0.02 (0.44)	−0.06 (0.40)^B^	−0.11 (0.38)	0.01 (0.40)
**Correlation of Function Rating to sEMG ratio:**				
**Pain History**				
**Transient**	0.28 (0.62)	0.43 (0.42)^A^	0.01 (0.69)	0.50 (0.34)^A^
**Chronic**	−0.04 (0.36)	−0.12 (0.45)^B^	0.04 (0.37)	0.00 (0.46)^B^
**Recurrent**	0.06 (0.40)	0.15 (0.35)	0.08 (0.33)	0.15 (0.40)
**Resolution of Pain**				
**Resolved**	0.27 (0.60)	0.42 (0.41)^A^	0.18 (0.62)	0.49 (0.32)^A^
**Partial**	0.16 (0.19)	0.08 (0.33)	0.20 (0.09)	0.19 (0.35)
**No Res**	0.02 (0.34)	0.02 (0.37)^B^	−0.02 (0.29)	−0.01 (0.41)^B^

A and B designations indicate significant differences in correlation coefficients between groupings.

Correlations for both pain and function to all dependent measures were generally stronger for the L5 ES site than the L2 site. The highest correlations of both pain and function were to EX-FR_L5_ for the Transient group in the pain history analysis, and for the Resolved group of the pain resolution analysis. MANOVA showed significant differences for both pain scores and function ratings, for both the FL-FR_L5_ and EX-FR_L5_ variables. For both variables Tukey's post hoc testing show significantly greater correlations for both pain and function in the Transient group than for the Chronic group. The Recurrent group did not significantly differ from the other two groups, but for all comparisons correlations were intermediate to the other two groups. No significant differences were observed for either flexion or extension ratios at the L2 level.

The groupings based on pain resolution produced similar trends among the correlation coefficients as did the pain history analysis. The MANOVA indicated significant differences in correlation to pain score for FL-FR_L5_. However, though the tendency was similar, the correlation to EX-FR_L5_ was not significant (p = 0.058). The correlation to function rating was significant for both FL-FR_L5_ and EX-FR_L5_. The Tukey's post hoc analysis showed significantly greater correlations for the Resolved group than for the Unresolved group. The correlations of the Partial resolution group were not significantly different than for either other group. No significant between-group differences were observed at the L2 level.

## Discussion

Significantly greater L5 sEMG amplitude was observed in the LBP group than the Control group at the time of the initial visit. This finding supports the observation generally reported throughout the literature of elevated sEMG amplitudes at full static flexion for those experiencing back pain [19]–[25], [27], [28], [30]. Disturbances of flexion relaxation response and how it changes over time may bear some relationship to reports of differing activation patterns of the lumbar musculature in individuals with nonspecific LBP. Different motor strategies in response to postural challenges in those with chronic LBP as compared to those without pain have been attributed to disturbances in sensory integration [35]. Other research suggests that those with chronic, nonspecifc LBP were less efficient and thus used more energy in controlling postural sway than healthy individuals [36]. Further research into how these motor behaviors might relate to the potential for developing chronicity could have implications for clinical management.

There were no significant between-group differences in the sEMG ratios between at the time of the first study session. This finding is contrary to a meta-analysis finding that FR ratios were often associated with lower FR ratios at full static flexion [18]. This may be due in large part to differences in study populations. The cohort of the present study had a varied pain experience as compared to the populations of the studies in the meta-analytic review, which generally had greater chronicity and functional disability. In addition to severity, other factors may explain differences between results of the present study and the reports of recovery of FR following rigorous functional restoration [29], [30] and exercise [29] interventions that involve strengthening of the back extensors. It is likely that training effects within the ES could be in part responsible for changes in activation patterns. Participants in the present study were not enrolled in any such programs.

A unique feature of the present study was the ability, by virtue of the repeated measures, to observe the interplay between a physiologic measure, and self-reported measures of pain and function over the course of the low back pain episode in a high functioning population. Comparing the strength of these correlations between participant groupings based on their pain history has allowed us to draw some inferences as to how an individual's perception or physiologic response to pain may vary relative to the activation patterns of the erector spinae. The post hoc tests showed that FL-FR_L5_ and EX-FR_L5_ ratios to both pain scores and functional rating were significantly greater for the Transient pain group than for those classified as having and chronic pain. The correlations between the ratios of flexion relaxation to both the forward bending and extension phases demonstrated a significantly stronger inverse relationship to pain report, and positive correlation with function, in the group experiencing transient symptoms than those with more chronic pain. In the Transient group, as pain decreased over the reporting period there was more often an increase in relative amplitude of both the flexion and extension phases relative to FR, reflecting a more “normal” behavior. This was generally not the case for the Chronic group. One explanation might be that because of either physiologic or perceptual changes, pain fear or avoidance behaviors can result in changes to lumbar movement patterns [37] that may not allow for as great a relative degree of relaxation of the trunk extensor musculature during full flexion, when ligamentous structures would normally bear the tissue loads. This speculation is strengthened by the fact that the correlations for the Recurrent group, who by definition are intermediate to the other groups, were also intermediate in response. The groupings based on the relative resolution of LBP over the reporting period produced similar trends. Here again, those who experienced a resolution of pain symptoms had a significantly stronger relationship between both pain and function to the flexion relaxation ratios than their cohort in whom LBP persisted to the end of the study. The speculations made above could apply as well to these results.

There was a trend worth noting in the strength of relationship between the sEMG ratios and the subjective reports dependent upon the level of erector spinae at which the sEMG was recorded. In general, correlations were weaker for the sEMG recorded at the L2 than the L5 level for both the Transient group in the pain history analysis, and in the Resolved group in the pain resolution analysis. Though the reason for this is not known, it has been reported that flexion relaxation occurs less consistently in progression in a cephalad (towards the head) direction [3]. This was particularly pronounced when comparing the correlations of the EX-FR_L2_ and EX-FR_L5_ ratios to both pain and function ratings in both group analyses.

The groupings based on degree of resolution of LBP during the course of the study suggest that in retrospect those whose symptoms resolved had a significantly stronger relationship between changes in the erector spinae activation patterns and their reports of pain and function changes. Participants who ultimately got better were, on some conscious or unconscious level, better able to relax their back extensors as their pain and function improved. One might speculate that as their muscle physiology returned to a more “normal” status, their perceived pain and reported functional status responded accordingly. Conversely, those whose symptoms and functional deficits persisted throughout the course of the study had significantly weaker relationships with muscle activation patterns, being unable to alter those patterns when at full trunk flexion in response to changes in pain, and ultimately function. This would lead to speculation that for those individuals, other psychosocial factors such as pain beliefs or anxiety [38] may be modulating their perceptions, if flexion relaxation is robust phenomenon. A similar logic can be applied to analysis based on grouping by pain history. This significant relationship, though not providing as strong a correlation between measures, also makes sense in that light. Those experiencing a first episode of LBP of less than six months duration were better able to relax the lower lumbar erector spinae as their pain resolved and function improved. In those facing more prolonged experience with low back pain a dissociation seems to develop between physical behaviors and perception of pain as well as function in daily living.

One limitation of the present study was that though the sample size was sufficient to observe statistically significant differences between participant groupings in some of the measures, power may not have been sufficient to observe others effects. Another potential limitation was the possibility of confounding with the treatment some participants were receiving. This risk was mitigated by having participants complete their ratings, and perform the experimental protocol prior to any treatment. Whether changes were secondary to the natural history, or secondary to treatment or other factors should not be of significant concern as the study investigated correlation of pain or function to EMG- derived variables, independent of potential cause.

The analysis of the relation of pain and function to the FR measures over time suggest that these relationships may have some utility in identifying those likely to progress to resolution of pain over a short period (eight weeks or less) from those who whose pain symptoms, and functional deficits persist. An important consideration in interpreting the results was that the participants more closely represented a cross-section of LBP in the community, as opposed to other studies with populations characterized by greater severity or functional deficits, making direct comparison of results difficult. Further study of the changes in the relationship of physiologic responses to LBP and function over time in such a cohort might help improve our understanding of the complex interplay of psychosocial factors and physical responses to back pain behaviors. Though it is not possible to draw conclusions about factors that may perpetuate back pain, improved understanding of this EMG phenomenon, and its relation to pain and function could ultimately provide measures useful in guiding clinical management.
